# Short-Term Effects of Overnight Orthokeratology on Corneal Sensitivity in Chinese Children and Adolescents

**DOI:** 10.1155/2018/6185919

**Published:** 2018-12-23

**Authors:** Wanqing Jin, Jiangping Ye, Jiafan Zhang, Yu Zhu, Frank Thorn, Ningning Liu, Ruzhi Deng

**Affiliations:** ^1^The Eye Hospital of Wenzhou Medical University, Wenzhou, China; ^2^School of Optometry and Ophthalmology and Eye Hospital, Wenzhou Medical University, Wenzhou, China; ^3^New England College of Optometry, Boston, MA, USA

## Abstract

**Purpose:**

To assess the effects of the 3-month period of orthokeratology (OK) treatment on corneal sensitivity in Chinese children and adolescents.

**Methods:**

Thirty subjects wore overnight OK lenses in both eyes for 3 months and were assessed at baseline, 1 day, 1 week, 1 month, and 3 months after the treatment. Changes in corneal sensitivity were measured by the Cochet–Bonnet (COBO) esthesiometer at the corneal apex and approximately 2 mm from the temporal limbus. Changes in refraction and corneal topography were also measured.

**Results:**

Central corneal sensitivity suffered a significant reduction within the first month of the OK treatment period but returned to the baseline level at three months (*F* = 3.009, *P*=0.039), while no statistically significant difference occurred in temporal sensitivity (*F* = 2.462, *P*=0.074). The baseline of central corneal sensitivity correlated with age (*r* = −0.369, *P*=0.045). A marked change in refraction (uncorrected visual acuity, *P* < 0.001; spherical equivalent, *P* < 0.001) and corneal topographical condition (mean keratometry reading, *P* < 0.001; eccentricity value, *P* < 0.001; Surface Regularity Index, *P* < 0.001) occurred, but none of these measurements were correlated with corneal sensitivity.

**Conclusions:**

A 3-month period OK treatment causes a reduction in central corneal sensitivity in Chinese children and adolescents but with a final recovery to the baseline level, which might be because neuronal adaptation occurred earlier in children and adolescents than in adults.

## 1. Introduction

Myopia is the most common ocular disorder in humans [1]. An epidemic of myopia has created a myopic prevalence in young adults in developed countries of east and southeast Asia of about 80–90%, with a 10–20% prevalence of high myopia [2]. Hence, it is important to suppress the progression of myopia. Overnight orthokeratology, using a special kind of rigid gas-permeable contact lens (RGPCL) with reverse-geometry design and higher DK material, is an increasingly popular clinical technique for temporarily correcting refractive errors and controlling myopia progression by reshaping the corneal contour. Previous studies have shown that OK lenses were also effective in slowing the progression of myopia in children [1, 3, 4]. In addition, over the last decade, the effects of OK lenses on corneal morphology, biomechanical properties [5–7], and the occurrence of complications [8] have been widely investigated to evaluate the safety of OK treatment, but its effect on corneal sensitivity still needs further study.

Normal corneal sensitivity plays a key role in maintaining the health of the ocular surface and physiologically reduces with advancing age [9–11] and more pigmented irises [11, 12]. Decreases in sensitivity can weaken the ability of the cornea to detect foreign bodies that could damage the ocular surface, as well as compromise the lacrimal functional unit, leading to a reduction in tear secretion and even to the dry eye [13–17]. It has already been clearly demonstrated that contact lens wear causes a reduction of corneal sensitivity regardless of the lens material and wearing modality [18–21]. However to date, only a few studies have shown the influence of OK lenses, the newly developed special contact lenses, on corneal sensitivity [22–26]. In a clinical trial conducted in adult patients wearing OK lenses for 3 months [26], Lum et al. observed that significant decreases in central and midperipheral corneal sensitivity occurred after 30 days of OK lens wear, with a further decrease seen in central corneal sensitivity at 90 days, but none in the midperiphery. What's more, Nombela-Palomo et al. noted a significant, but modest, decrease in central corneal sensitivity at 1-month OK treatment on adult patients [24]. However, subsequent research conducted by the same group found that corneal sensitivity values kept amazingly stable after one year of OK treatment when compared with baseline. A possible explanation for this stabilization could be neuronal adaptation to the pressure exerted by the lens which would have occurred at sometime between three months and one year of treatment [25].

Today, there are more than 1.5 million orthokeratology patients in China [27], and the majority of them are children and adolescents with a burning desire to control their myopia progression. Since almost all of the existing OK studies related to corneal sensitivity were based on non-Chinese adult subjects, different with Chinese children in age and iris color [22–26], our study was designed to prospectively discover and analyze the changes produced in corneal sensitivity on Chinese children and adolescents undergoing OK treatment for 3 months.

## 2. Materials and Methods

This was a prospective longitudinal study of 3 months' duration. The study protocol was approved by the Ethics Committee of the Affiliated Eye Hospital of Wenzhou Medical University, China (No. 2016–6) and adhered to the tenets of the Declaration of Helsinki. Subjects were examined in 5 separate visits: baseline, 1 day, 1 week, 1 month, and 3 months after starting OK treatment. In each visit, several measurements were performed on both eyes between 2 and 4 hours after removing the lenses (a 5-minute interval occurred between every two measurements): in order, uncorrected and corrected visual acuity, subjective refraction, corneal topography, and corneal sensitivity.

### 2.1. Subjects

Thirty-three subjects with a desire for refractive reduction therapy by orthokeratology were recruited for the study based on the following inclusion criteria: myopia up to –5.00 diopters (D) with astigmatism of 1.50 D or less, best-corrected visual acuity (BCVA) of 20/20 or better, age between 8 and 18 years, and having no previous history of orthokeratology lens wear or having stopped wearing them for at least 1 month immediately before being recruited. Subjects with ocular or systemic disorders, including herpes simplex keratitis, diabetes, myasthenia gravis, and those with a history of ocular disease or previous ocular surgery were excluded from the study. Written informed consent was obtained from all study participants after the risks and benefits of orthokeratology lens wear, and the measurement techniques had been explained to the subjects and a parent in detail.

### 2.2. Lenses

Alpha overnight orthokeratology (Alpha Corporation, Japan), which is the reverse-geometry rigid contact lens used in this study, has a special design (base curve, reverse curve, alignment curve, and peripheral curve). The properties of the lenses are presented in Table 1. The lenses were fit according to the manufacturer's guidelines and were provided without charge. All subjects were recommended to wear the lenses for at least 7 continuous hours every night and remove them immediately when they wake up.

### 2.3. Subjective Refraction and Corneal Topography

Refractive error was estimated by NIDEK RT-3100 intelligent refractor (NIDEK Co., Ltd, Japan) without cycloplegia and refined by a subjective refraction. Changes in corneal topography were monitored by measuring corneal eccentricity (*E* value), the Surface Regularity Index (SRI), and mean keratometry (Km) using Medmont E300 corneal topographer (Medmont Pty Ltd, Melbourne, Australia).

### 2.4. Corneal Sensitivity Measurement

We used a COBO esthesiometer (Luneau, Paris, France) to obtain sensitivity measurements at the corneal apex and approximately 2 mm from the temporal limbus. The COBO esthesiometer consists of a 0.12 mm diameter nylon monofilament of variable length (0–60 mm) that applies pressure on the cornea in the range of 11 to 200 mg/0.0113 mm^2^. The filament was positioned perpendicular to the corneal surface until it made a contact with the surface. To take the central measurements, subjects were asked to stare at a point 5 m ahead. For the midperipheral measurements, subjects redirected their fixation towards a second point located horizontally to the left or right of the central point. The procedure was started by positioning the filament at its maximal length (60 mm), which corresponds to the lowest possible pressure. If a subject did not feel the filament at 60 mm, the filament length was reduced at 5 mm intervals until the subject felt the filament. The threshold of corneal sensation is defined by the length of the longest filament necessary to obtain at least 2 positive responses in 3 times of stimulations.

### 2.5. Statistical Analysis

For all variables, data from the right eye only was considered. The Kolmogorov–Smirnov test was used to determine the distribution of the obtained data (*P* > 0.05). Parametric and nonparametric statistical tests were then used to analyze normally distributed and nonnormally distributed data, respectively. Changes in the variables examined produced between baseline and each follow-up session were compared by repeated measures analysis of variance (RM-ANOVA). When comparing baseline measurements between the two groups with a different lens-wearing history, nonnormally distributed data were analyzed using the Mann–Whitney *U* test (IBM SPSS statistics version 22; IBM, NY). A reported value of *P* < 0.05 denotes a statistical significance. Correlations between corneal sensitivity and baseline of other parameters in the study were determined using Pearson's correlation for normally distributed data and Spearman's rank order correlation for nonnormally distributed data.

## 3. Results

The final study sample consisted of 30 right eyes of 30 subjects aged 11.07 ± 2.13 years, including 14 boys and 16 girls (in the 33 recruited subjects, 2 withdrew from the study because they left the city for personal reasons and 1 for mild keratitis). 8 of the 30 subjects had experienced the orthokeratology for about 1 year but stopped it for at least 1 month before enrolling in the study. None of the OK lens wearers who completed the 3-month treatment suffered any adverse events, and no abnormalities of the eyes were detected by slit-lamp microscopy. The main parameters recorded in the 3-month study are all provided in Table 2.

### 3.1. Central Corneal Sensitivity Suffered a Modest Reduction but Recovered to the Baseline Level during the 3-Month Treatment

**Figure 1:**
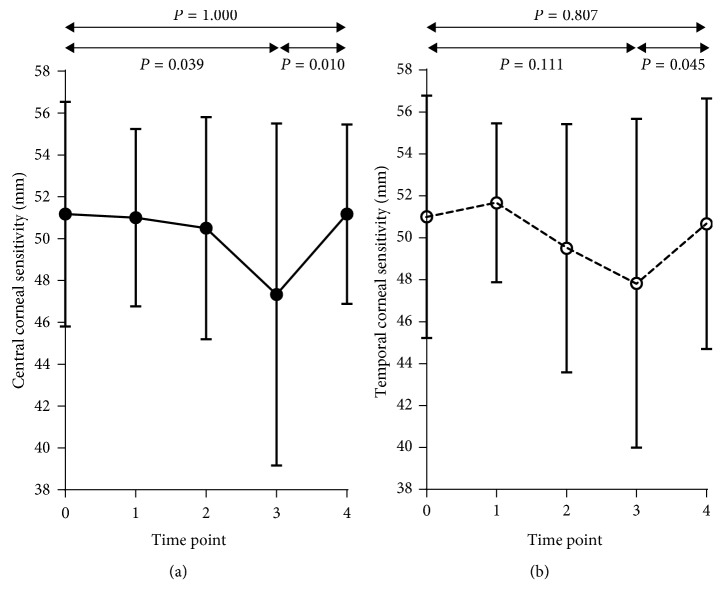
Changes in corneal sensitivity at both central cornea (a) and temporal cornea (b) during the 3-month OK treatment period. *X* axis shows the time points (0 = baseline, 1 = 1 day after treatment, 2 = 1 week after treatment, 3 = 1 month after treatment, and 4 = 3 months after treatment), *Y* axis shows the corneal sensitivity (mm) means ± SD. Solid line represents changes in central corneal sensitivity, and dashed line represents changes in temporal corneal sensitivity. There was a statistically significant difference between the baseline of central corneal sensitivity and the 1-month time period, but no difference between baseline and 3 months.

**Figure 2:**
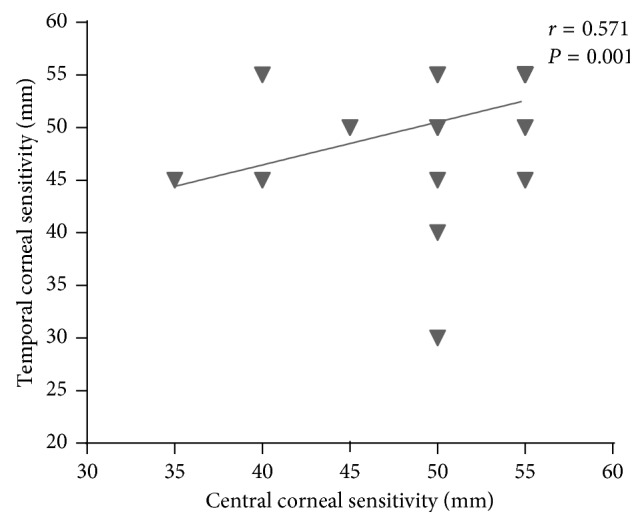
Correlation between the baselines of central corneal sensitivity and temporal corneal sensitivity. *X* axis is central corneal sensitivity (mm), and *Y* axis is temporal corneal sensitivity (mm). Central corneal sensitivity correlated positively with temporal corneal sensitivity (*r* = 0.571, *P*=0.001).

The significant differences observed from baseline to 3 months after treatment were as follows: (1) central corneal sensitivity suffered a reduction in 1 month after treatment (*F* = 3.009, *P*=0.039) but returned to the baseline level at the final visit (Figure 1(a)), while changes in temporal corneal sensitivity were not statistically significant (*F* = 2.462, *P*=0.074) but showed a trend similar to that of central corneal sensitivity (Figure 1(b)); (2) a significant reduction occurred in SE (*F* = 106.499, *P* < 0.001), Km (*F* = 39.960, *P* < 0.001), and *E* value (*F* = 18.124, *P* < 0.001), while a remarkable improvement was found in UCVA (*F* = 67.776, *P* < 0.001) and SRI (*F* = 21.260, *P* < 0.001); (3) BCVA remained stable (*F* = 1.374, *P*=0.257); and (4) mean of the baseline of central corneal sensitivity of the 8 subjects worn OK lenses previously was 48.75 ± 5.18 mm, while that of the remaining 22 ones was 52.05 ± 5.27 mm, and a statistically insignificant difference occurred between them (*P*=0.081).

### 3.2. Baseline of Central Corneal Sensitivity Correlated Negatively with Age

**Figure 3:**
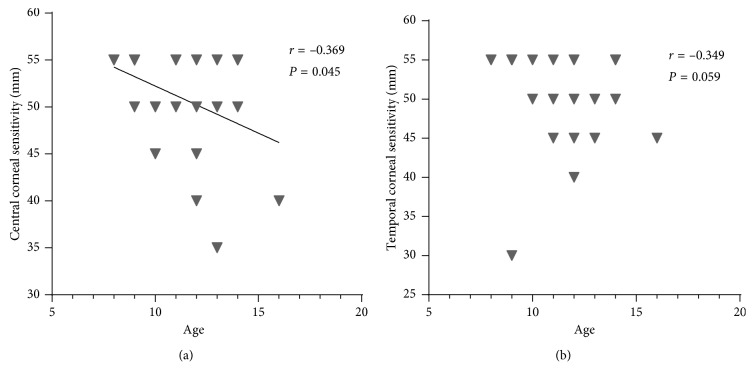
Correlation between corneal sensitivity baseline and age. (a) *X* axis is age, and *Y* axis is central corneal sensitivity (mm). Central corneal sensitivity correlated negatively with age (*r* = −0.369, *P*=0.045). (b) *X* axis is age, and *Y* axis is temporal corneal sensitivity (mm). No correlation occurred between them (*r* = −0.349, *P*=0.059).

Correlations between the baselines of corneal sensitivity and other measurements are as follows: (1) correlation between central corneal sensitivity and temporal corneal sensitivity (*r* = 0.571, *P*=0.001) (Figure 2); (2) correlations between central corneal sensitivity and age (*r* = −0.369, *P*=0.045) (Figure 3(a)) and between temporal corneal sensitivity and age (*r* = −0.349, *P*=0.059) (Figure 3(b)). This shows central corneal sensitivity decreased with increasing age; (3) no correlation between central corneal sensitivity and refractive changes (UCVA *r* = 0.027, *P*=0.886; SE *r* = 0.091, *P*=0.632) or corneal topography (Km: *r* = 0.177, *P*=0.350 and SRI: *r* = 0.095, *P*=0.619); and (4) no correlation between temporal corneal sensitivity and refractive changes (UCVA: *r* = 0.042, *P*=0.825 and SE: *r* = −0.171, *P*=0.367) or corneal topography (Km: *r* = 0.181, *P*=0.339 and SRI: *r* = 0.019, *P*=0.919).

### 3.3. Discussion

The goal of this study was to demonstrate the effects of a 3-month OK treatment on corneal sensitivity in Chinese children and adolescents, and it tries to offer a reasonable explanation of the findings.

The means of corneal sensitivity values recorded here were within the normal limits of previous articles, though slightly lower than those reported for normal corneas [9]. However, Lum et al. found that central corneal sensitivity continued to decrease in his 3-month OK treatment without recovery. He ascribed the reductions to sensory adaptation rather than morphological changes in the corneal nerve fibers [26]. In addition, Nombela-Palomo et al. observed a continuous modest reduction of central corneal sensitivity in a 1-month OK treatment period which she thought was caused by a functional change in the corneal nerves rather than the morphological change [24]. In a continuation of this study, she discovered an unexpected consistency in central corneal sensitivity after one year of orthokeratology relative to that before treatment and attributed it to an undefined neuronal adaptation. Her studies showed this recovery occurred between one month and one year but she combined her data with Lum's data and concluded that the recovery occurred between three months and one year [25]. The changes in central corneal sensitivity during the first month in our study are basically the same as those in previous studies, and neuronal adaptation was thought to be the main contributor to this change [24, 26]. However, our findings show that the recovery of corneal sensitivity occurred within 3 months of OK treatment. This is not consistent with Nombela-Palomo's conclusion [25]. Neuronal adaptation is the time-dependent modulation of sensory responses following sequential stimuli and thought to be a consequence of synaptic plasticity [28]. Synaptic plasticity, defined as activity-dependent modifications in the efficacy and strength of synaptic transmission of preexisting synapses [29], reduces with advancing age [30, 31], probably due to age-related changes of significant receptors [32, 33] and alterations in the expression of genes related to them [31]. Given that the subjects in our study differed in age from the previous studies, it can be suggested that neuronal adaptation is affected by age because it occurs earlier in children and adolescents than in adults. Further investigations are needed to confirm this hypothesis.

In our study, although the means of central corneal sensitivity values of 8 subjects who already have OK lenses wearing experience seemed slightly lower than that of 22 subjects without wearing experience, we found there was no statistically significant difference between them. Moreover, previous studies have shown that central corneal sensitivity can be restored to the baseline level after stopping wearing OK lenses for 1 month (or even rebound to the higher level) [25, 34]. Therefore, we included the 8 subjects together in the analysis.

None of the temporal corneal sensitivity changes produced in any of the groups was significant though highly correlated with central corneal sensitivity. This finding was consistent with that reported in previous studies, which found that the changes in corneal sensitivity occurred only in the central cornea while the midperipheral cornea remained unaffected [23, 24].

The reduction in central corneal sensitivity with advancing age detected in this study has been reported in previous literature [10, 11]. A rapid onset of refractive and corneal topographical change was also shown in our study. Of course, this is the purpose of wearing OK lenses and is consistent with previous OK studies [26, 35, 36]. It reveals the efficacy of OK in correcting refractive errors. However, no correlations were detected between these optical changes and corneal sensitivity.

There are certain limitations in the present study. First, according to the inherent characteristics, the COBO esthesiometer, a positive response can be affected by the subject's attitude and apprehension [24]. However, it is the traditional standard ocular surface sensitivity assessment, commonly used in clinical practice and in research, and its repeatability of corneal sensitivity measurement has been proved to be good [9, 37]. Throughout this study, corneal sensitivity was measured by the same well-trained operator in the same room to ensure the reliability and accuracy. Second, if values of corneal sensitivity at 1 year after OK treatment were measured, we would know more about the long-term effect of OK on corneal sensitivity. Third, the results would be more convincing if the sample size was enlarged.

In conclusion, we have demonstrated that Chinese children and adolescents suffered a reduction in central corneal sensitivity, but it returned to the baseline level by the end of the 3-month OK treatment period, as measured by the COBO. This means wearing OK lenses for a short time may have no significant effect on corneal sensitivity in children and adolescents because of the strong corneal nerve repairing ability.

## Figures and Tables

**Table 1:** Properties of alpha orthokeratology.

Property	Content
Material	Boston EM
Refractive index	1.422
Oxygen transmission coefficient (Dk)	104 × 10ˉ^11^ (cm^2^/s) (mLO_2_/mL × mmHg)
Visible light transmittance	81.0%
Wetting angle	35°
Optical center thickness (mm)	0.22

**Table 2:** Parameter values previous to OK treatment and changes produced in the 3-month OK treatment.

Parameter	Baseline	1 day	1 week	1 month	3 months	*F*	*P* ^*∗*^
	(Mean ± SD)				
UCVA (logMAR)	0.63 ± 0.34	0.33 ± 0.21^a^	0.07 ± 0.15^ab^	0.02 ± 0.12^abc^	0.01 ± 0.13^abc^	67.776	<0.001
BCVA (logMAR)	−0.03 ± 0.04	−0.03 ± 0.04	−0.05 ± 0.04^b^	−0.04 ± 0.05	−0.04 ± 0.07	1.374	0.257
SE (D)	−2.77 ± 1.04	−1.31 ± 0.81^a^	−0.33 ± 0.69^ab^	−0.13 ± 0.56^ab^	−0.18 ± 0.44^ab^	106.499	<0.001
Km (D)	43.16 ± 1.53	42.52 ± 1.37^a^	41.90 ± 1.49^ab^	41.80 ± 1.54^ab^	41.79 ± 1.55^ab^	39.960	<0.001
SRI	0.55 ± 0.26	1.03 ± 0.25^a^	1.12 ± 0.25^ab^	1.12 ± 0.32^a^	1.03 ± 0.36^a^	21.260	<0.001
E value	0.62 ± 0.09	0.40 ± 0.15^a^	0.36 ± 0.19^a^	0.38 ± 0.17^a^	0.37 ± 0.18^a^	18.124	<0.001
CCS (mm)	51.17 ± 5.36	51.00 ± 4.24	50.50 ± 5.31	47.33 ± 8.17^abc^	51.17 ± 4.29^d^	3.009	0.039
TCS (mm)	51.00 ± 5.78	51.67 ± 3.79	49.50 ± 5.92^b^	47.83 ± 7.84^b^	50.67 ± 5.98^d^	2.462	0.074

SD, standard deviation; UCVA, uncorrected visual acuity; BCVA, best-corrected visual acuity; SE, spherical equivalent; Km, mean keratometry reading; SRI, Surface Regularity Index; *E* value, eccentricity value; CCS, central corneal sensitivity; TCS, temporal corneal sensitivity. ^a^Statistically significantly different from baseline at *P* < 0.05; ^b^statistically significantly different from 1 day after treatment at *P* < 0.05; ^c^statistically significantly different from 1 week after treatment at *P* < 0.05; ^d^statistically significantly different from 1 month after treatment at *P* < 0.05.

## Data Availability

All the data related to this article are available from the corresponding author on reasonable request.
